# Circular RNAs as Biomarkers and Therapeutic Targets in Diabetic Cardiovascular Complications

**DOI:** 10.1002/edm2.70149

**Published:** 2025-12-17

**Authors:** Shreya Singh Beniwal, Yujin Jeong, Akash Rawat, Mahmoud Einieh, Kashyapi Patil, Rafael Everton Assunção Ribeiro da Costa, Pulkit Saini, Kamal Yousef Ghazal, Abhijeet Kumar, Ayush Dwivedi, Anuja Anil Mandavkar

**Affiliations:** ^1^ Lady Hardinge Medical College New Delhi India; ^2^ Icahn School of Medicine at Mount Sinai/Elmhurst New York New York USA; ^3^ Himalayan Institute of Medical Sciences Swami Rama Himalayan University Dehradun India; ^4^ University of Debrecen Debrecen Hungary; ^5^ Krishna Vishwa Vidyapeeth Malkapur, Karad Maharashtra India; ^6^ Cidade Universitária “Zeferino Vaz,” Barão Geraldo, Campinas State University of Campinas (UNICAMP) São Paulo Brazil; ^7^ Sri Devaraj Urs Medical College Kolar Karnataka India; ^8^ King Fahad Medical City Riyadh Saudi Arabia; ^9^ Netaji Subhas Medical College and Hospital Patna Bihar India; ^10^ Danylo Halytsky Lviv National Medical University Lviv Ukraine; ^11^ Vilasrao Deshmukh Government Medical College Latur Maharashtra India

**Keywords:** biomarkers, cardiovascular, circular RNAs, complications, diabetes, therapeutic

## Abstract

**Background:**

The global prevalence of diabetes mellitus (DM) is rising rapidly and is projected to reach unprecedented levels by 2035 and 2050, with a disproportionate burden among elderly populations and in low‐ and middle‐income countries. Diabetes‐related cardiovascular complications remain a leading cause of morbidity and mortality despite advances in glycaemic control and pharmacotherapy. There is an urgent need for novel molecular biomarkers and therapeutic targets to improve early detection, risk stratification, and personalised management.

**Methods:**

A comprehensive narrative review of the current literature was conducted to summarise emerging evidence on the biological roles of circular RNAs (circRNAs) in diabetes and its cardiovascular complications. Published experimental, translational, and clinical studies investigating circRNA expression, mechanisms of action, and therapeutic potential were critically analysed.

**Results:**

CircRNAs have emerged as key regulators in the pathophysiology of diabetes‐associated cardiovascular disorders, including diabetic cardiomyopathy, endothelial dysfunction, and vascular inflammation, as well as related microvascular complications. Mechanistically, circRNAs act through diverse pathways such as microRNA sponging, modulation of gene transcription, interaction with RNA‐binding proteins, and regulation of cellular processes including apoptosis, fibrosis, oxidative stress, and inflammation. Their covalently closed structure confers exceptional stability, while their tissue‐ and disease‐specific expression profiles support their utility as sensitive biomarkers for early diagnosis, prognosis, and therapeutic monitoring. Advances in synthetic circRNA design further highlight their promise as novel therapeutic agents, although challenges related to delivery efficiency, specificity, and off‐target effects remain.

**Conclusions:**

CircRNAs represent a promising class of biomarkers and therapeutic targets in diabetes‐related cardiovascular complications. Their stability, specificity, and functional versatility position them as attractive tools for precision medicine approaches in diabetes care. Further mechanistic studies and well‐designed clinical investigations are essential to translate circRNA‐based diagnostics and therapeutics into clinical practice.

## Graphical abstract

This graphic abstract elaborates on the recent mechanism of circular RNAs (circRNAs) in the pathogenesis, diagnosis and treatment of diabetic cardiovascular complications. Diabetes mellitus induces oxidative/inflammatory stress, followed by endothelial dysfunction and leads to micro‐/macro‐vascular complications. By virtue of their covalently closed‐loop structures, circRNAs modulate these pathways mainly by functioning as miRNA sponges, transcriptional and protein regulators. Certain circRNAs are associated with diabetic cardiomyopathy, nephropathy, neuropathy and retinopathy. Being of stable structure, tissue‐specific distribution and differential expression, circRNAs have great potential as non‐invasive biomarkers and therapeutic targets. The emerging technologies for delivering these circRNAs (e.g., siRNA, nanoparticles, exosomes) also endorse the feasibility of their applications.

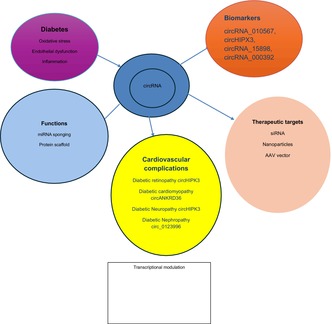

**Abbreviations**
AAVs: adeno‐associated viruses
CAN: cardiovascular autonomic neuropathy
CircRNAs: circular RNAs
ciRNA: circular intronic RNAs
CVD: cardiovascular disease
DCM: diabetic cardiomyopathy
DM: diabetes mellitus
DN: diabetic nephropathy
DNP: diabetic neuropathy
DR: diabetic retinopathy
DRG: dorsal root ganglia
ECM: extracellular matrix
EIciRNA: exon‐intron containing circular RNA
lncRNA: long non coding RNA
MIAT: myocardial infarction‐associated transcript
miRNA: microRNA
mRNA: messenger RNA
ncRNA: non coding RNA
NO: nitric oxide
PPARγ: peroxisome proliferator‐activated receptor
RBPs: RNA binding proteins
RNAi: RNA interference
RPE: retinal pigment epithelium
siRNA: small interfering RNA
vWF: von Willebrand factor

## 1 Introduction

**FIGURE 1:**
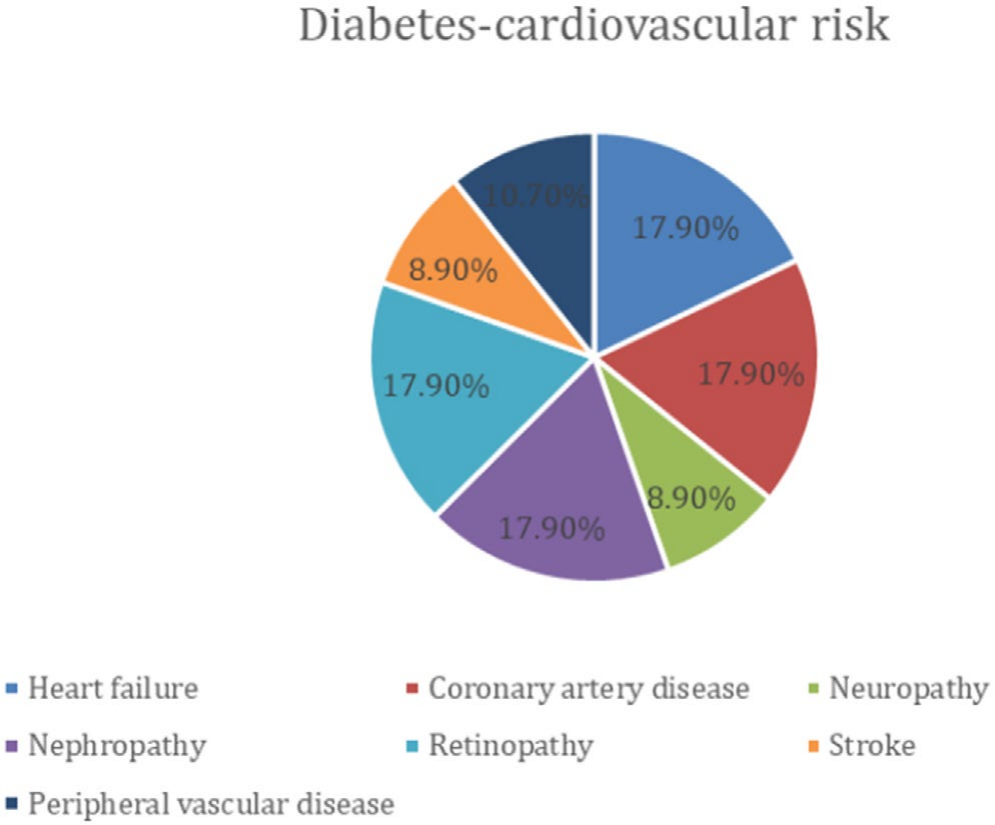
The image depicts prevalence of complications in diabetes [2].

**FIGURE 2:**
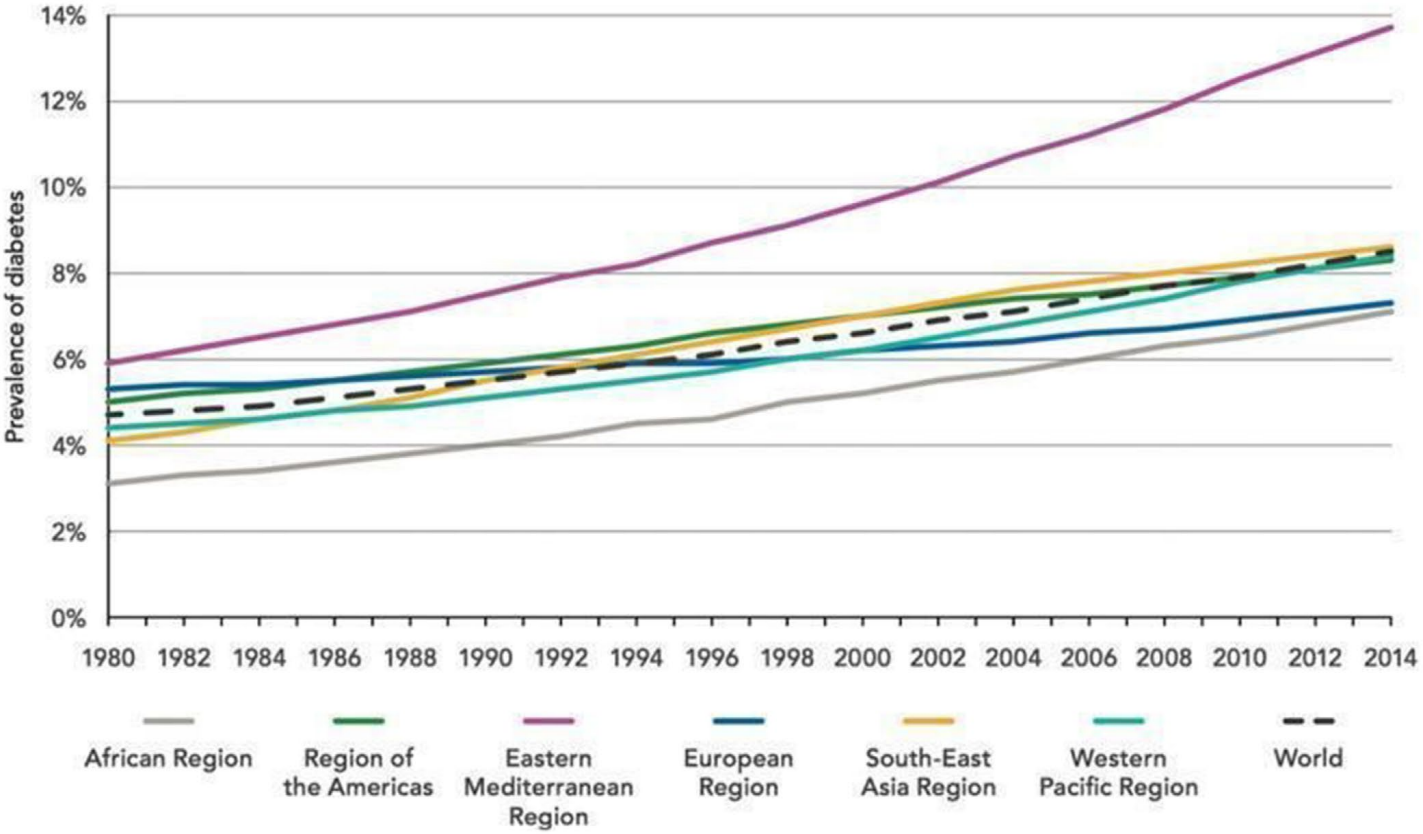
Trends in prevalence of diabetes region‐wise, 1980–2014, by WHO [3].

The global incidence of diabetes mellitus (DM) has reached alarming proportions, representing one of the most serious public health challenges of the 21st century. It is not only a metabolic disorder but a complex, multisystem disease with profound cardiovascular consequences. According to the International Diabetes Federation, the number of individuals affected is projected to surpass 1.3 billion by 2050, with the steepest rise occurring in low‐ and middle‐income countries, particularly in regions such as South Asia, North Africa and the Middle East [1, 2]. Figure 1 represents the prevalence of various micro‐ and macrovascular complications of diabetes, while Figure 2 depicts regional trends in diabetes prevalence from 1980 to 2014 (WHO) [4, 5]. This epidemiological panorama underscores the urgent need for novel biomarkers and therapeutic strategies capable of mitigating the burden of diabetes‐related cardiovascular complications.

Diabetes‐associated cardiovascular disease (CVD) encompasses a spectrum of clinical entities including atherosclerosis, coronary artery disease (CAD), diabetic cardiomyopathy (DCM) and heart failure, which collectively account for more than half of all diabetes‐related deaths [6, 7]. Chronic hyperglycemia, insulin resistance and dyslipidemia promote oxidative stress, endothelial dysfunction, inflammation and myocardial fibrosis, leading to irreversible structural and functional cardiac damage [8, 9]. Conventional biomarkers such as HbA1c or C‐reactive protein lack the sensitivity to detect early cardiovascular alterations, highlighting the unmet need for molecular tools with superior specificity and prognostic power [10].

In this context, circular RNAs (circRNAs) have emerged as promising candidates for both diagnostic and therapeutic innovation [11, 12]. These molecules represent a unique subclass of noncoding RNAs characterised by covalently closed loop structures that confer exceptional stability, resistance to exonucleases and tissue‐ and disease‐specific expression patterns [3, 13]. Unlike linear RNAs or microRNAs (miRNAs), circRNAs exhibit longer half‐lives, compartmentalised expression and multifunctional regulatory capacities, including the ability to act as microRNA sponges, scaffolds for RNA‐binding proteins (RBPs) and modulators of transcription and translation [14, 15, 16].

Recent studies have begun to elucidate the involvement of specific circRNAs in key pathogenic pathways of diabetic CVD, such as inflammation, oxidative stress and vascular remodelling [17, 18, 19]. For instance, dysregulated expression of circHIPK3, circ_000203 and circ_010567 has been linked to myocardial fibrosis, endothelial dysfunction and smooth‐muscle proliferation—hallmarks of DCM [20, 21, 22]. Beyond their mechanistic roles, circRNAs display measurable alterations in plasma and tissue samples, making them accessible targets for liquid‐biopsy‐based diagnostics [23].

Despite these advances, our understanding of the translational potential of circRNAs remains incomplete. Challenges persist regarding the standardisation of detection techniques, validation of candidate molecules in large patient cohorts, and optimisation of delivery systems for circRNA‐based therapeutics [24, 25]. Nevertheless, the expanding evidence base suggests that circRNAs could transform the clinical management of diabetes‐related cardiovascular complications by enabling early detection, risk stratification and individualised therapy [26, 27].

Therefore, this review aims to provide a comprehensive synthesis of current evidence on the biogenesis, mechanisms and functional relevance of circRNAs, with a special emphasis on their roles as biomarkers and therapeutic targets in diabetic cardiovascular complications. Furthermore, it will explore ongoing technological and translational challenges, highlighting future directions necessary to bring circRNA‐based diagnostics and therapies closer to clinical practice.

## 2 Overview of Diabetes‐Related Cardiovascular Complications

Diabetes as an affliction is rapidly increasing due to the growing prevalence of obesity and lifestyle changes, which increase the risk of developing the disease [10, 11]. Type 2 DM is the most common form of diabetes, accounting for approximately 90%–95% of all diagnosed cases worldwide [12]. As shown in Figure 2, the global prevalence of diabetes has risen continuously across regions from 1980 to 2014, reflecting a persistent epidemiological trend that parallels the increasing burden of CVD.

Diabetes mellitus is a well‐established independent risk factor for the development of both microvascular and macrovascular complications, making it one of the leading causes of morbidity and mortality worldwide. Among these, CVD represents the predominant cause of death, accounting for nearly half of all diabetes‐related fatalities. Insulin resistance and hyperglycemia, characteristic of both type 1 and type 2 diabetes, are recognised as the major contributors to cardiovascular complications [13]. Through complex metabolic pathways, chronic hyperglycemia induces endothelial dysfunction, inflammation, oxidative stress and lipid abnormalities, which together promote atherosclerosis, myocardial fibrosis and impaired cardiac function.

Types of cardiovascular complications associated with diabetes:

### 2.1 Diabetic Cardiomyopathy

Diabetic cardiomyopathy is defined as the presence of structural or functional myocardial abnormalities in patients with diabetes, occurring independently of other cardiac risk factors such as hypertension or CAD [14]. Though diabetes is commonly associated with hypertension and CAD, which can lead to cardiomyopathy, it has been demonstrated that diabetes itself directly contributes to myocardial injury [15, 16]. Type 2 diabetes is strongly linked to obesity, which increases circulating free fatty acids and leads to myocardial lipotoxicity. This process enhances oxidative stress through excessive mitochondrial fatty acid oxidation, ultimately resulting in diastolic dysfunction and interstitial fibrosis [16].

### 2.2 Cardiovascular Autonomic Neuropathy (CAN)

Cardiovascular autonomic neuropathy (CAN) is responsible for a nearly fivefold increase in cardiovascular mortality among diabetic patients. Its pathogenesis involves autonomic nervous system dysregulation triggered by chronic hyperglycemia and microvascular ischemia. Inadequate glucose control leads to loss of nitric oxide bioavailability, accumulation of free radicals within Schwann cells and neuronal degeneration, all of which predispose diabetic individuals to renal disease, stroke and sudden cardiac death [17].

### 2.3 Coronary Artery Disease

Diabetes is one of the major risk factors for the development of CAD, and its incidence is significantly higher among diabetic populations compared with nondiabetic individuals [18]. The pro‐thrombotic state characteristic of diabetes, driven by endothelial dysfunction, increased expression of von Willebrand factor (vWF) and platelet activation, accelerates atherogenesis and plaque instability [19]. These changes lead to premature CAD and worsen outcomes following acute coronary events.

### 2.4 Stroke

The risk of cerebrovascular accidents is also markedly elevated in diabetic patients. Both ischemic and hemorrhagic strokes are more frequent and may even occur at younger ages compared to nondiabetic populations [20]. Uncontrolled diabetes acts as a risk factor for both stroke subtypes [21]. The vascular pathology in diabetes often results in lacunar strokes, presenting clinically with limb weakness and dysarthria due to small‐vessel occlusion.

### 2.5 Impact of Diabetes on Patient and Health Care System

Beyond the biological mechanisms, diabetes‐related cardiovascular complications impose an enormous socioeconomic burden. Individuals living with diabetes experience a marked reduction in quality of life, increased hospitalisation rates and higher mortality, while healthcare systems face escalating costs due to chronic disease management and disability [22]. Understanding the molecular pathways that underlie these complications—particularly those regulated by circRNAs—offers not only mechanistic insight but also the prospect of developing precision‐based diagnostics and therapeutics that could alleviate this growing public health crisis.

Collectively, these cardiovascular sequelae illustrate the systemic impact of diabetes and the intricate interplay between metabolic, inflammatory and vascular pathways. This integrated perspective establishes the biological foundation upon which circRNAs act as pivotal regulatory molecules in diabetes‐related CVD; a topic further elaborated in the next section of this review.

## 3 Circular RNAs: An Introduction

Circular RNA was first identified from pathogenic plant viroids in 1976 [23]. Subsequently, circular RNA molecules were observed in animal systems, including the murine respirovirus (Sendai virus) [24]. Since their discovery, circRNAs have evolved from being considered molecular curiosities to being recognised as highly functional regulatory molecules in eukaryotic cells. They are now understood to represent a unique subclass of noncoding RNAs that regulate gene expression at multiple levels: transcriptional, post‐transcriptional and translational, playing critical roles in cellular homeostasis, disease development and intercellular communication.

CircRNAs are characterised by a covalently closed circular structure, generated through a process known as back‐splicing, in which a downstream 5′ splice donor site is joined to an upstream 3′ splice acceptor site. This circular configuration distinguishes them from linear RNAs, as it confers resistance to exonuclease degradation and results in a significantly longer half‐life, making circRNAs exceptionally stable molecules within cells and extracellular fluids [25, 26].

**FIGURE 3:**
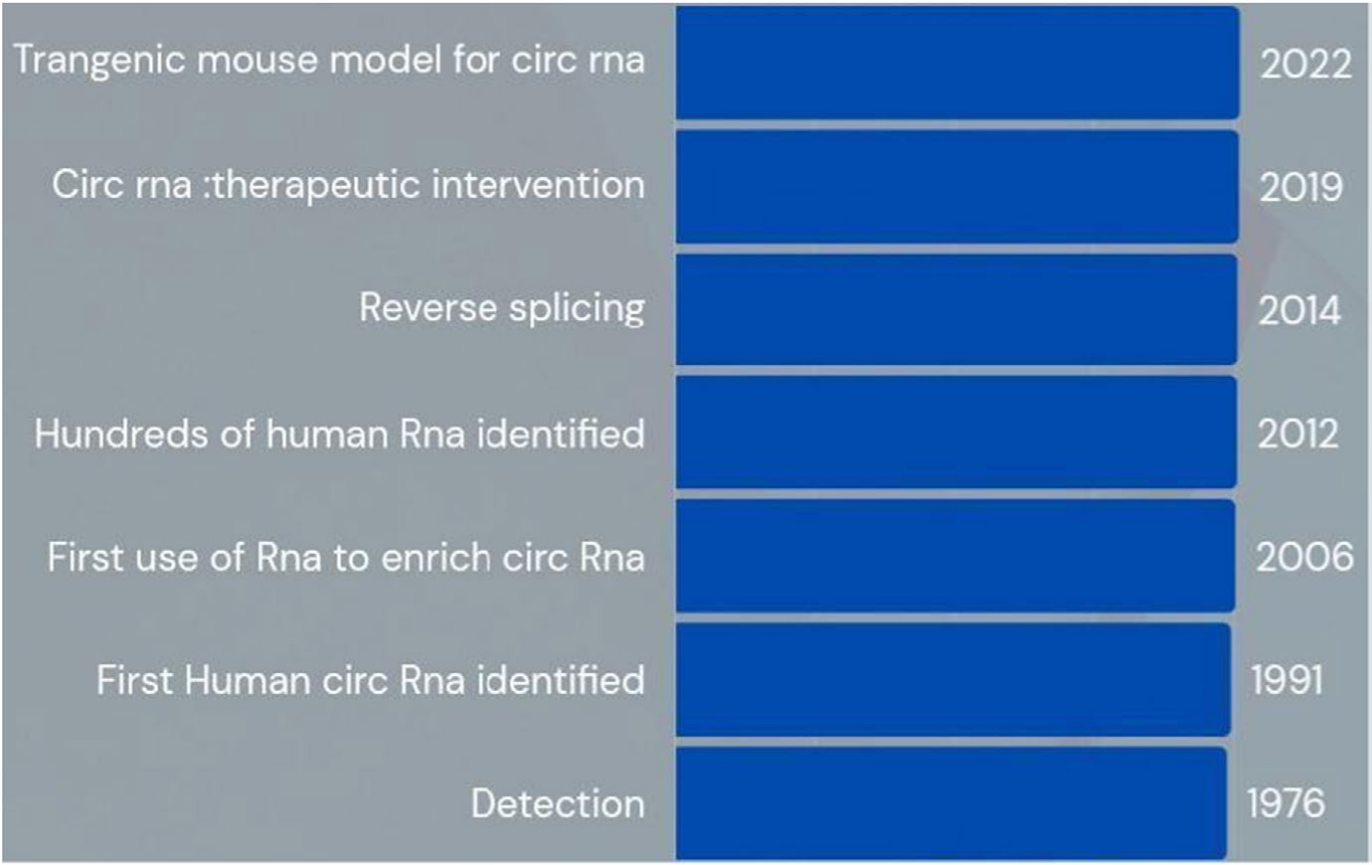
This image depicts the evolution of circRNA [26].

Thousands of circRNAs have been identified across mammalian species, including humans, and exhibit tissue‐, cell type‐ and developmental stage–specific expression patterns [27, 28, 29, 30, 31, 32]. Such specificity suggests that circRNAs are not transcriptional byproducts but are under tight regulatory control, often participating in key physiological and pathological processes. Figure 3 provides a schematic representation of the evolution of circRNA.

Functionally, circRNAs serve as versatile molecular regulators. They can act as microRNA (miRNA) sponges, binding to specific miRNAs and preventing them from suppressing their target messenger RNAs (mRNAs). For example, CiRS‐7 (also known as CDR1as) contains multiple binding sites for miR‐7, effectively sequestering it and modulating the expression of miR‐7–regulated genes [31]. Additionally, circRNAs interact with RBPs, influencing transcriptional activity or serving as scaffolds for ribonucleoprotein complex assembly. Some circRNAs can even be translated into functional peptides via internal ribosome entry sites (IRES), thereby contributing directly to protein synthesis [32, 33, 34].

Beyond their molecular functions, circRNAs have emerged as key modulators of cellular signalling in health and disease. Their remarkable stability and abundance in extracellular vesicles, including exosomes and plasma, make them promising biomarkers for diagnostic and prognostic applications [35]. Furthermore, their ability to regulate processes such as inflammation, oxidative stress, apoptosis and fibrosis directly links them to the molecular pathways underlying diabetes‐related cardiovascular complications [36, 37, 38].

**FIGURE 4:**
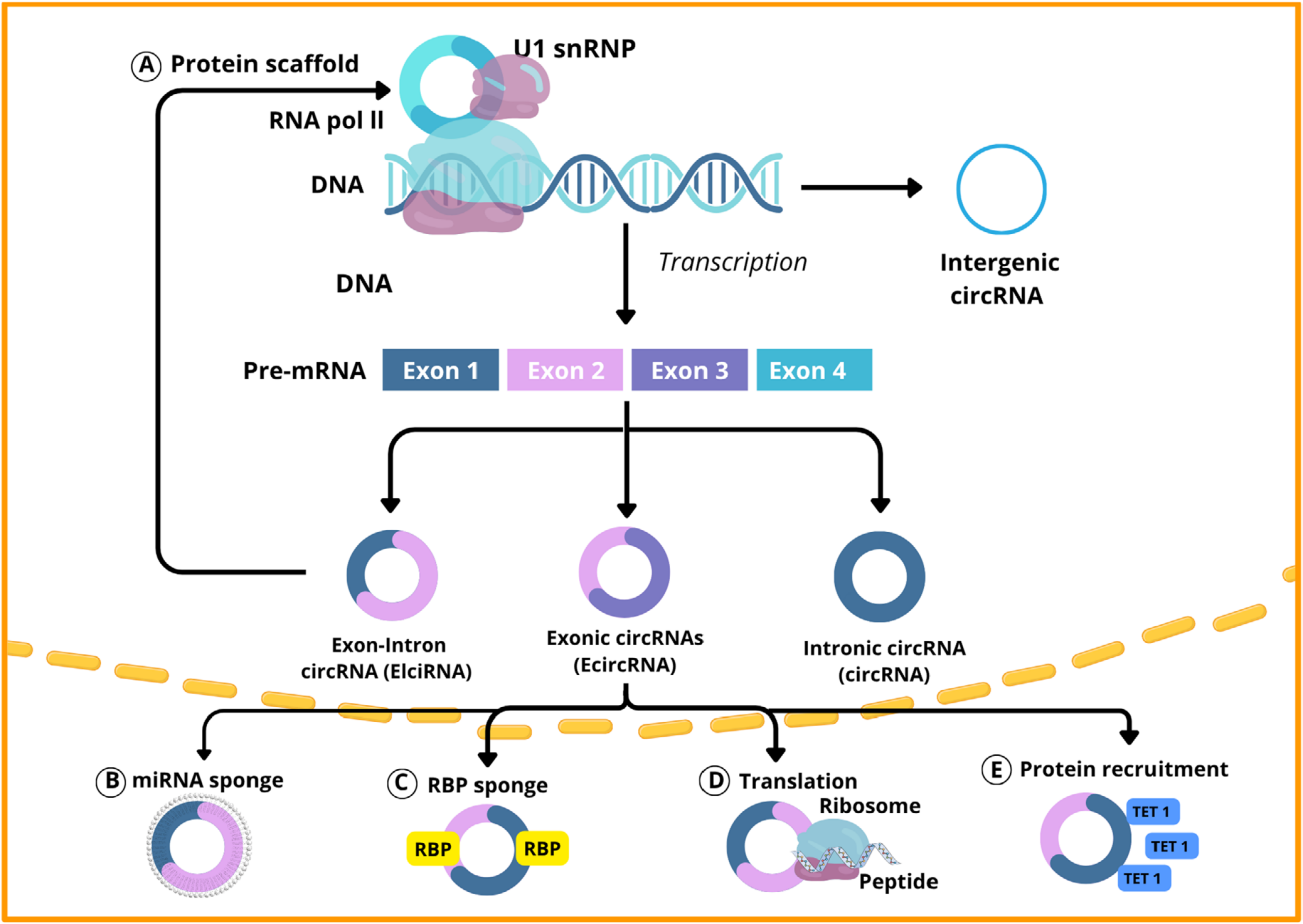
Formation and functions of circular RNAs. (A) Back‐splicing driven by intronic complementarity, RBPs or exon skipping generates exonic, exon–intron or intronic circRNAs. (B–E) Functional mechanisms include miRNA and RBP sponging, modulation of transcription, cap‐independent translation and protein recruitment. Adapted from Wang et al., 2021 [39, 40, 41].

Thus, circRNAs are not merely structural curiosities but pivotal regulators of cardiovascular homeostasis and pathology. Their biogenesis and multifaceted functions (Figure 4) provide a foundation for understanding how they modulate key molecular networks, including those involved in inflammation, oxidative stress and vascular remodelling, which are further explored in the subsequent sections of this review.

### 3.1 Biogenesis and Functions of CircRNAs

Circular RNAs (circRNAs) are a distinct class of endogenous RNA molecules characterised by a covalently closed‐loop structure that lacks both 5′ caps and 3′ poly(A) tails. This circular conformation results from a back‐splicing event, in which a downstream splice donor site is joined to an upstream splice acceptor site via a phosphodiester bond [29]. The absence of free ends renders circRNAs resistant to exonuclease‐mediated degradation, thereby conferring a much longer half‐life and higher stability than their linear RNA counterparts. This structural stability allows circRNAs to persist in diverse tissues, plasma and exosomes, positioning them as potential biomarkers in physiological and pathological conditions.

CircRNAs are generated from pre‐mRNAs transcribed by RNA polymerase II through the canonical spliceosome machinery. The process of back‐splicing can occur via two major pathways:

- Lariat‐driven circularization: during canonical splicing, exons are skipped and the remaining lariat intermediate undergoes circularization.
- Intron‐pairing–driven circularization: complementary sequences within flanking introns, such as inverted ALU repeats, align and bring splice sites into proximity, facilitating circularization [30, 31, 32].

The spliceosome complex plays a central role by mediating the covalent linkage between the donor and acceptor sites. In addition, RBPs act as regulatory trans‐factors that promote or inhibit circularization. Among them, *Quaking (QKI)* and *Muscleblind‐like (MBL)* are well‐characterised examples: both bind to specific intronic motifs and stabilise the interaction between flanking introns, thereby enhancing circRNA formation [33, 34]. Conversely, certain RBPs can also repress back‐splicing by competing for binding sites or destabilising intronic complementarity.

Mammalian‐wide interspersed repeat elements further modulate this process, influencing the efficiency and tissue specificity of circRNA generation. The coordination between cis‐elements (complementary intronic sequences) and trans‐factors (RBPs) therefore determines which transcripts undergo circularisation and in which cellular context.

### 3.2 Classification of CircRNAs

CircRNAs can be broadly classified into three main types according to their composition and origin [31]:

**Table:** 

Exonic circRNAs (ecircRNAs)	Derived exclusively from exons and mainly localised in the cytoplasm, where they frequently function as post‐transcriptional regulators
Exon–intron circRNAs (EIciRNAs)	Containing both exonic and intronic sequences; typically nuclear, often regulating the transcription of their parental genes
Circular intronic RNAs (ciRNAs)	Composed solely of intron sequences that escape debranching after canonical splicing, predominantly localised in the nucleus

### 3.3 Function of Circular RNAs

#### 3.3.1 Regulation of Gene Expression

CircRNAs function as key modulators of gene expression at multiple levels. One of the most studied mechanisms is microRNA (miRNA) sponging, whereby circRNAs sequester miRNAs and prevent them from repressing their mRNA targets. The classical example is CiRS‐7 (also known as CDR1as), which contains over 60 binding sites for miR‐7, thereby modulating gene networks involved in neuronal and metabolic regulation [35]. Through this mechanism, circRNAs indirectly upregulate miRNA‐regulated genes.

#### 3.3.2 Interaction With RNA‐Binding Proteins

CircRNAs also bind to RBPs, influencing their availability, localization or function. For example, circFOXO3 acts as a scaffold for CDK2 and p21, forming an inhibitory complex that regulates cell cycle progression. Similarly, other circRNAs interact with splicing factors or transcriptional regulators, thereby contributing to post‐transcriptional and epigenetic modulation.

#### 3.3.3 Transcriptional and Translational Regulation

Certain nuclear circRNAs, particularly EIciRNAs and ciRNAs, interact with RNA polymerase II and promote transcription of their parental genes. Conversely, some cytoplasmic circRNAs contain IRESand open reading frames (ORFs), enabling cap‐independent translation into functional micropeptides—an emerging mechanism that broadens their regulatory potential [36].

#### 3.3.4 Functional Relevance in Disease

Beyond their molecular versatility, circRNAs play central roles in the pathophysiology of complex diseases, including metabolic and cardiovascular disorders. Their stability and tissue specificity enable them to act as molecular rheostats that fine‐tune pathways related to inflammation, oxidative stress, apoptosis and fibrosis—mechanisms that are pivotal in the development of diabetes‐related cardiovascular complications [37, 38].

### 3.4 Biological and Clinical Implications

The multifaceted nature of circRNAs provides a foundation for their diagnostic, prognostic and therapeutic potential. Their stability in biofluids makes them ideal candidates for liquid‐biopsy–based biomarkers, while their regulatory capacity opens avenues for therapeutic modulation using antisense oligonucleotides or RNA interference.

Understanding the mechanisms governing circRNA biogenesis and function is therefore essential for elucidating their contribution to cardiovascular dysfunction in diabetes. These principles form the basis for the subsequent sections of this review, which explore their mechanistic links, biomarker potential and translational applications.

## 4 CircRNAs As Biomarkers in Diabetes‐Related Cardiovascular Complications

### 4.1 CircRNAs as Potential Biomarkers

Biomarkers are fundamental tools for the diagnosis, prognosis and monitoring of chronic diseases. Their clinical relevance depends on stability, specificity, sensitivity and reproducibility. In this context, circular RNAs (circRNAs) have gained attention as next‐generation RNA biomarkers due to their closed‐loop structure, which grants exceptional resistance to exonucleases and makes them detectable in a wide range of biofluids, including plasma, serum and urine [41, 42].

Unlike linear RNAs, circRNAs exhibit tissue‐ and disease‐specific expression patterns, enabling them to reflect disease progression and organ‐specific injury. Their abundance in exosomes further enhances their clinical potential, as these vesicles protect RNA molecules from degradation during circulation.

**FIGURE 5:**
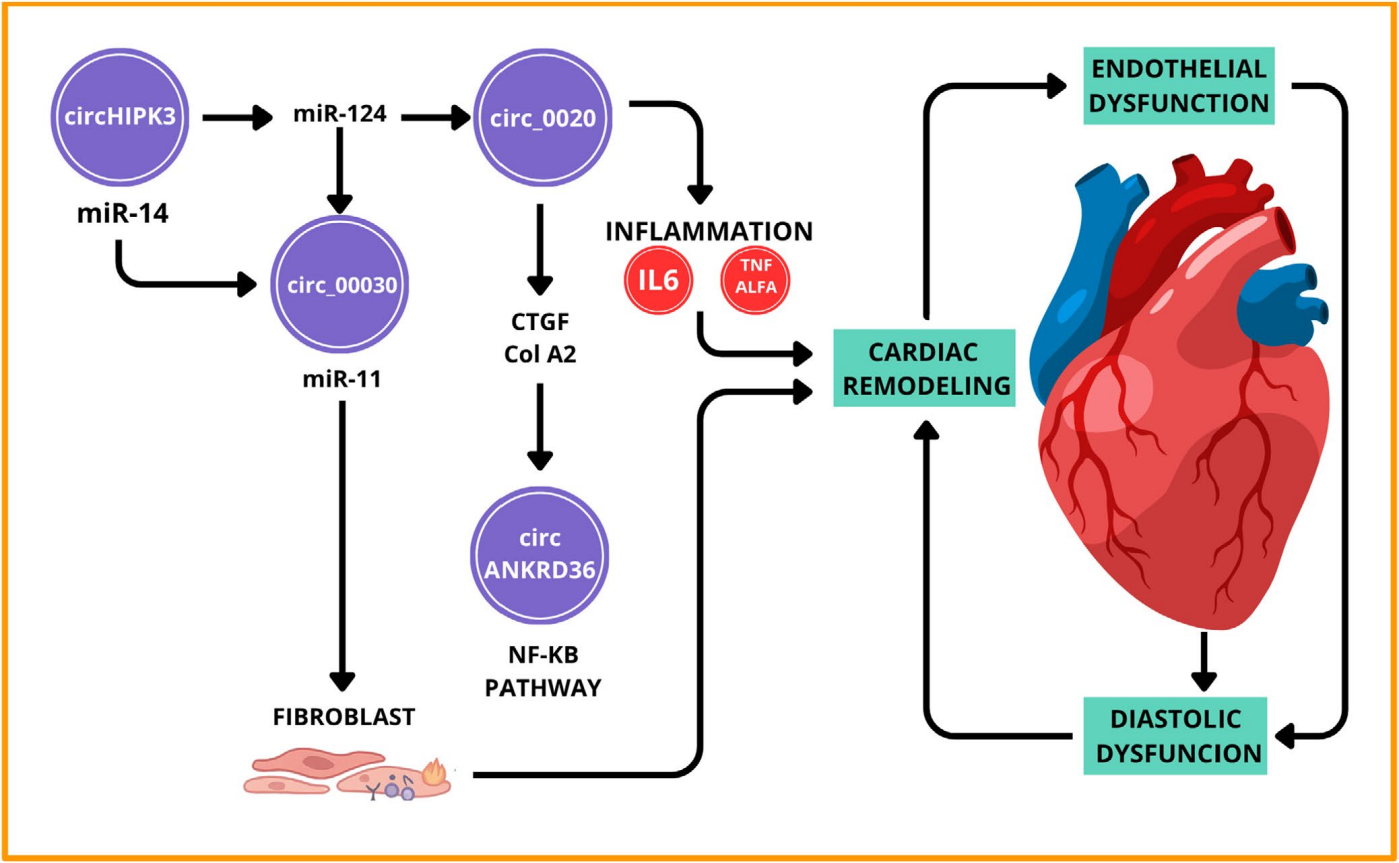
Illustration of major circular RNAs (circRNAs) modulating cardiac remodelling under hyperglycemic stress. circHIPK3, circ_000203 and circ_010567 enhance the expression of fibrogenic mediators such as connective tissue growth factor (CTGF) and collagen type II alpha 1 (COL2A1) through activation of the TGF‐β1/Smad and NF‐κB signalling cascades, resulting in myocardial fibrosis and inflammation. circANKRD36 contributes to the upregulation of IL‐6 and TNF‐α, promoting inflammatory remodelling and endothelial dysfunction. Collectively, these circRNA–miRNA–mRNA regulatory axes drive maladaptive cardiac hypertrophy and diastolic dysfunction characteristic of DCM [45].

Several studies have demonstrated the diagnostic promise of circRNAs in diabetes and its complications. For instance, circ_0123996 and circ_0000285 have been associated with podocyte autophagy in diabetic nephropathy (DN), whereas circHIPK3 and circZNF609 are markedly upregulated in retinal endothelial cells from patients with diabetic retinopathy (DR) [43, 44, 45]. Increased plasma levels of circHIPK3 have also been detected in individuals with diabetic neuropathic pain, reinforcing its potential as a biomarker of neural injury in metabolic disease [46, 47]. Figure 5 illustrates key circRNAs and their interaction with major signalling pathways implicated in the pathogenesis of DCM, including NF‐κB activation, oxidative stress and fibrosis.

### 4.2 CircRNAs in Cardiovascular Complications of Diabetes

Within the cardiovascular system, several circRNAs have been implicated in the development of DCMand atherosclerosis, where they modulate inflammation, oxidative stress and fibrosis. For example:

- circHIPK3 enhances endothelial proliferation by sponging miR‐124 and upregulating ICAM‐1, contributing to vascular remodelling.
- circ_000203 and circ_010567 are overexpressed in cardiac fibroblasts under hyperglycemic conditions, promoting myocardial fibrosis via the TGF‐β1/Smad and NF‐κB pathways.
- circ_0076631 (circANKRD36) has been associated with inflammatory activation in monocytes of diabetic patients and correlates with glycemic control and C‐reactive protein levels [48, 49, 50].

**FIGURE 6:**
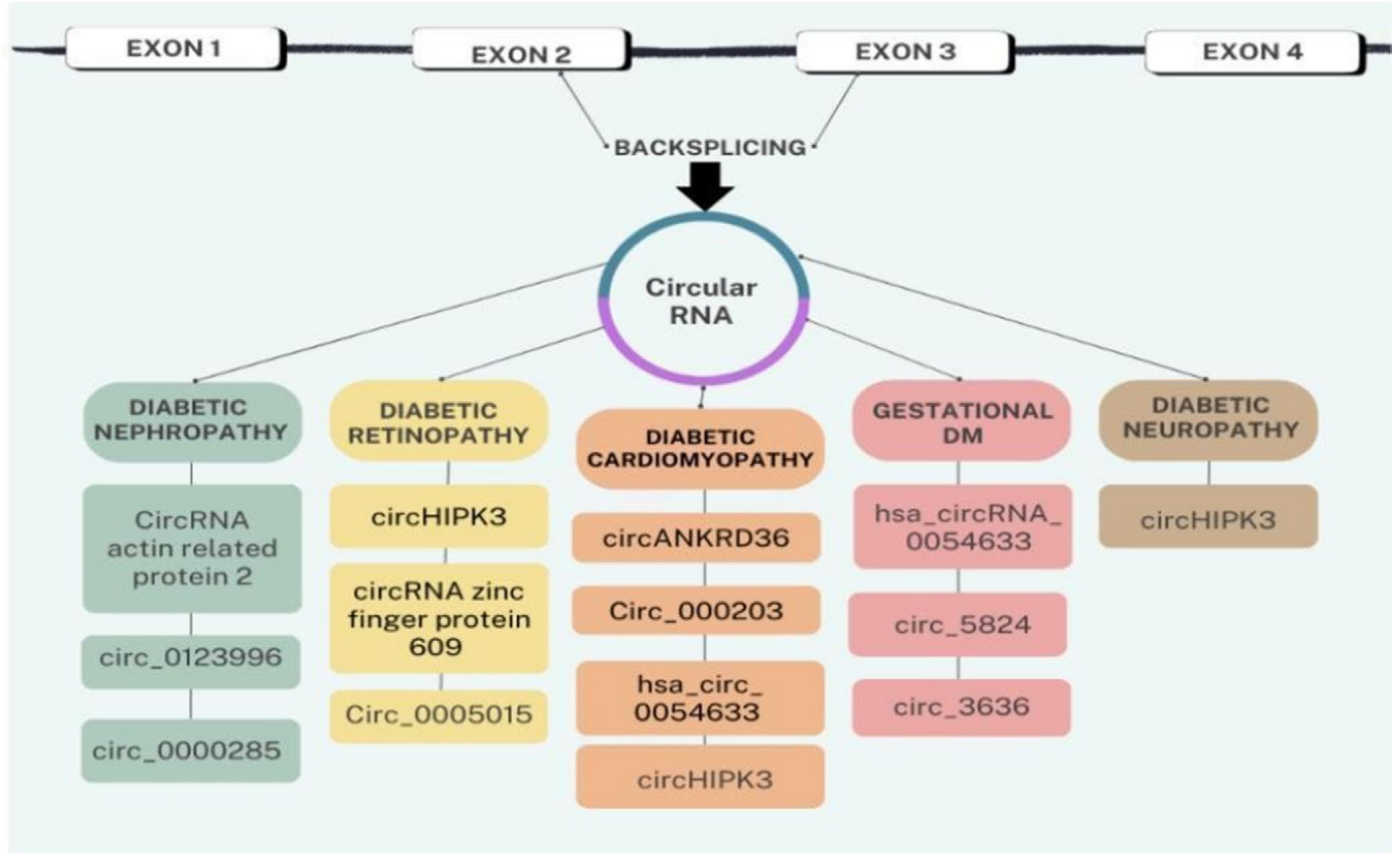
Representative circRNAs associated with microvascular and macrovascular complications of diabetes mellitus [43, 44, 45].

**FIGURE 7:**
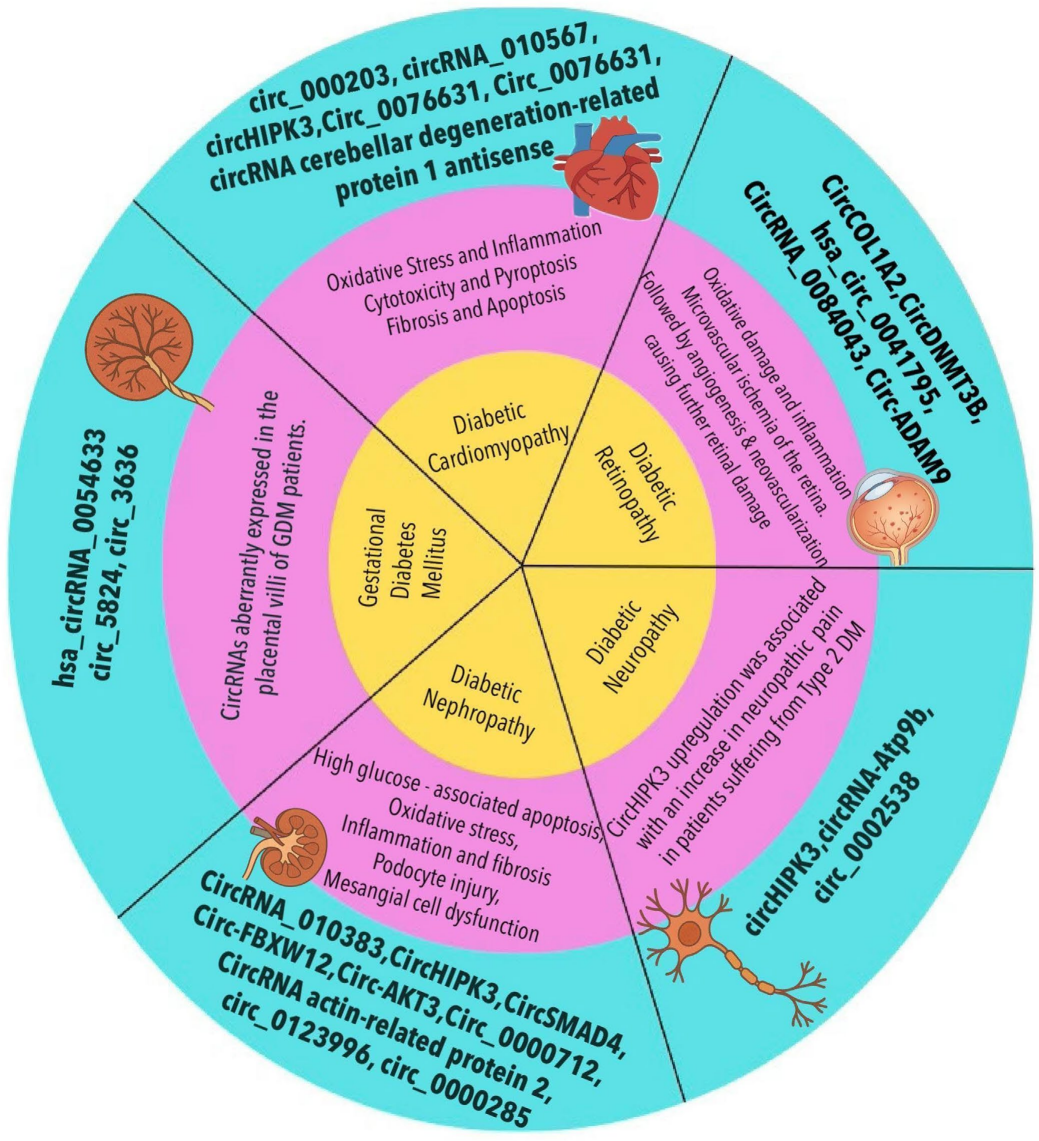
Representing circRNAs and their role in the pathogenesis of the cardiovascular complications of diabetes mellitus [44, 45, 46, 47, 50, 51, 52, 53, 54, 55, 56, 57, 58].

These findings suggest that specific circRNAs mirror disease activity and tissue damage, serving as both diagnostic and prognostic markers in diabetes‐related cardiovascular complications. Importantly, several studies report diagnostic accuracies (AUC values above 0.80) for circRNAs such as circ_000203 and circHIPK3 in identifying DCM, indicating promising clinical translatability. Figures 6 and 7 bellow illustrate the various circular RNAs involved in both microvascular and macrovascular complications of diabetes.

## 5 CircRNAs in the Pathophysiology of Diabetes‐Related

### 5.1 Cardiovascular Complications

Circular RNAs (circRNAs) are increasingly recognised as pivotal regulators in the molecular network linking DM to its micro‐ and macrovascular complications. Their ability to modulate inflammation, oxidative stress, angiogenesis, apoptosis and fibrosis positions them as critical mediators of endothelial dysfunction and myocardial remodelling in hyperglycemic conditions [52, 53].

### 5.2 Diabetic Retinopathy

Diabetic retinopathy provides a valuable framework to understand the microvascular injury mechanisms that also operate in the heart and peripheral vasculature. CircRNAs produced by endothelial cells, pericytes and retinal pigment epithelial (RPE) cells regulate angiogenic and inflammatory cascades that are equally relevant to systemic vascular health.

For example, circCOL1A2, derived from retinal microvascular endothelial cells, is markedly upregulated in patients with DR and influences extracellular‐matrix deposition and TGF‐β signalling, leading to capillary thickening and fibrosis [54]. Conversely, circDNMT3B, also of endothelial origin, exerts protective effects by suppressing aberrant VEGF‐driven neovascularization, thereby attenuating retinal ischemia [59].

In pericytes, circCPWWP2A and circEhmt1 preserve endothelial integrity and barrier function under hyperglycemic stress, preventing capillary dropout and ischemia [60]. Within RPE cells, several circRNAs act as regulators of cell death pathways:

- Downregulation of hsa_circ_0041795 and circ_0084043 protects against oxidative damage and apoptosis;
- Upregulation of circADAM9 promotes RPE apoptosis, while circPSEN1 downregulation mitigates ferroptosis, reducing inflammatory cytokine release [59, 60, 61, 62, 63, 64].

Together, these observations reveal how circRNAs coordinate angiogenesis, oxidative stress and inflammation in the diabetic retina, processes that mirror systemic endothelial dysfunction, myocardial fibrosis and vascular remodelling in diabetes‐related CVD.

### 5.3 Diabetic Nephropathy (DN)

As per a recent study, circEIF4G2 has been shown to increase renal fibrosis through the miR‐218/SERBP1 pathway, while overexpression of circRNA_010383 has been shown to inhibit renal fibrosis and proteinuria in a mouse model [65]. CircHIPK3 knockdown in a rat model led to a decrease in the mRNA of fibrogenic factors such as COL1, TGF‐beta and fibronectin in mesangial cells, leading to a decrease in cellular proliferation. CircRNA_15698 has been shown to increase the deposition of extracellular matrix (ECM) in a mouse model, while circSMAD4 upregulation, circ‐FBXW12 downregulation and circ‐AKT3 upregulation inhibit ECM deposition [66, 67, 68, 69, 70]. CircSMAD4 and Circ‐AKT3 upregulation was also shown to reduce inflammation and apoptosis [68]. Circ_0000712 increases high glucose‐associated apoptosis, oxidative stress, inflammation and fibrosis in DN by upregulating SOX6 expression [71]. Circ_0000285 has been shown to increase podocyte injury via MAPK6 activation in mouse podocytes [72]. CircTAOK1 acting through the SMAD3 axis and Circ‐ACTR2 acting through the HMGA2 axis have been shown to cause mesangial cell dysfunction and increase the progression of DN [73, 74].

### 5.4 Diabetic Neuropathy

Diabetic neuropathy (DNP) arises from chronic hyperglycemia‐induced microvascular ischemia, oxidative damage and axonal degeneration. Transcriptomic profiling of sciatic nerves and dorsal root ganglia (DRGs) in diabetic mice identified over 11,000 circRNAs, among which 15 were significantly dysregulated, including circRNA.4614, which showed marked upregulation in diabetic models [75].

Functionally, circHIPK3 is elevated in patients with diabetic neuropathic pain, where it modulates miR‐124 and miR‐30 targets, promoting neuroinflammation and hyperexcitability of sensory neurons [76]. These data indicate that circRNAs may influence neuronal‐glial communication and pain sensitization through oxidative and inflammatory cascades, though clinical validation remains limited.

### 5.5 Diabetic Cardiomyopathy: Cardiac Fibrosis and Apoptosis Regulation

Diabetic cardiomyopathy is driven by metabolic stress, mitochondrial dysfunction and interstitial fibrosis, and circRNAs have been shown to modulate these processes at multiple levels. circRNA_000203 and circHIPK3 are upregulated in diabetic myocardium, promoting fibrotic remodelling through activation of the TGF‐β1/Smad2/3 pathway and repression of antifibrotic miRNAs [77, 78, 79].

Interestingly, other studies suggest a dual role for circHIPK3: its upregulation can also decrease PTEN expression, thereby activating the Akt pathway, suppressing apoptosis and preserving cardiomyocyte viability under hyperglycemic conditions [80]. These apparently contradictory results highlight the cell‐ and context‐dependent functions of circRNAs in cardiac homeostasis.

**FIGURE 8:**
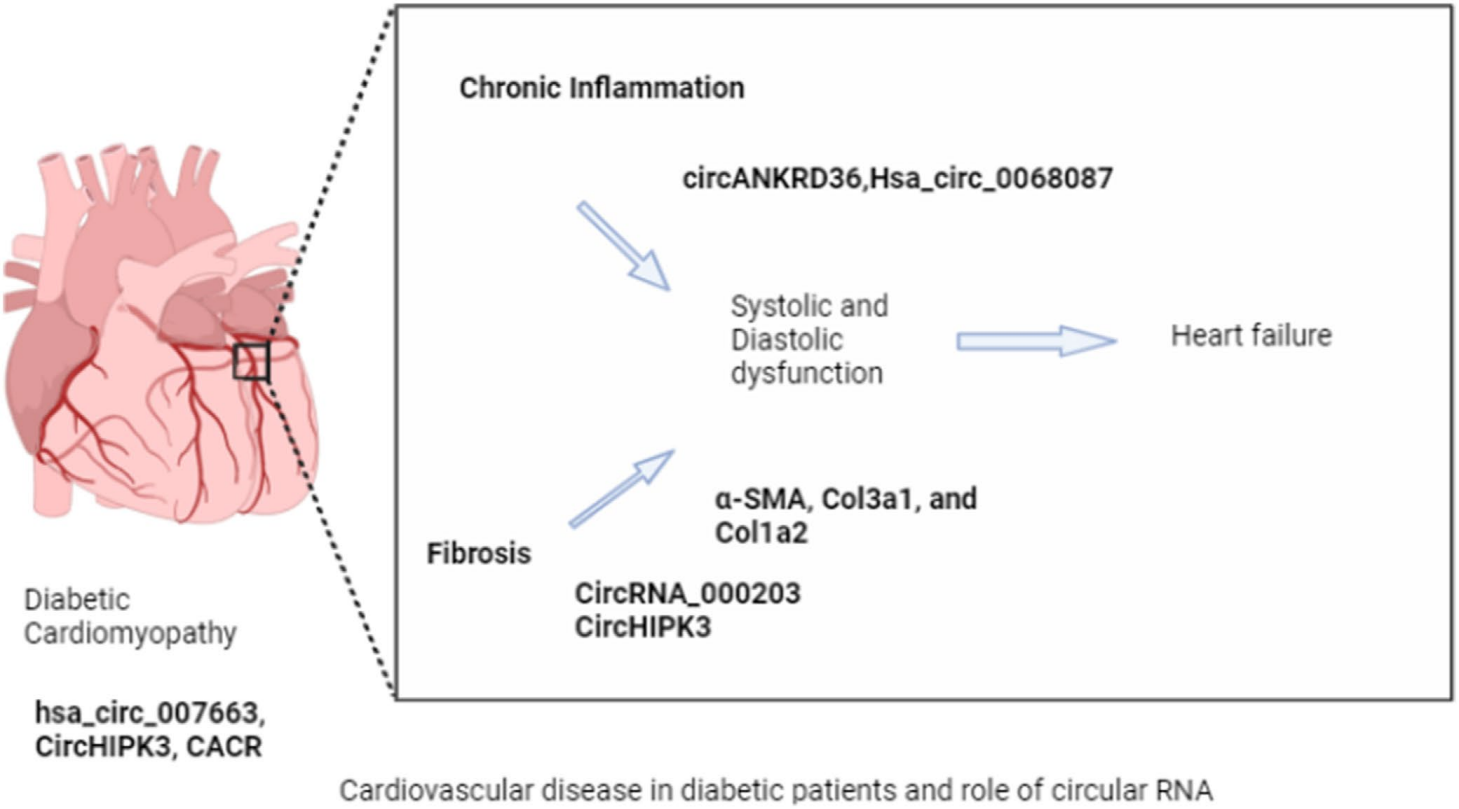
The following figure illustrates the pathophysiology of diabetic cardiomyopathy and various circular RNAs upregulated in the same disease process [77, 78, 79, 80].

Figure 8 illustrates the key circRNAs and signalling pathways implicated in DCM, emphasising the balance between pro‐fibrotic and cytoprotective mechanisms.

### 5.6 Cardiovascular Disease in Diabetes: Endothelial Dysfunction and Inflammation

**FIGURE 9:**
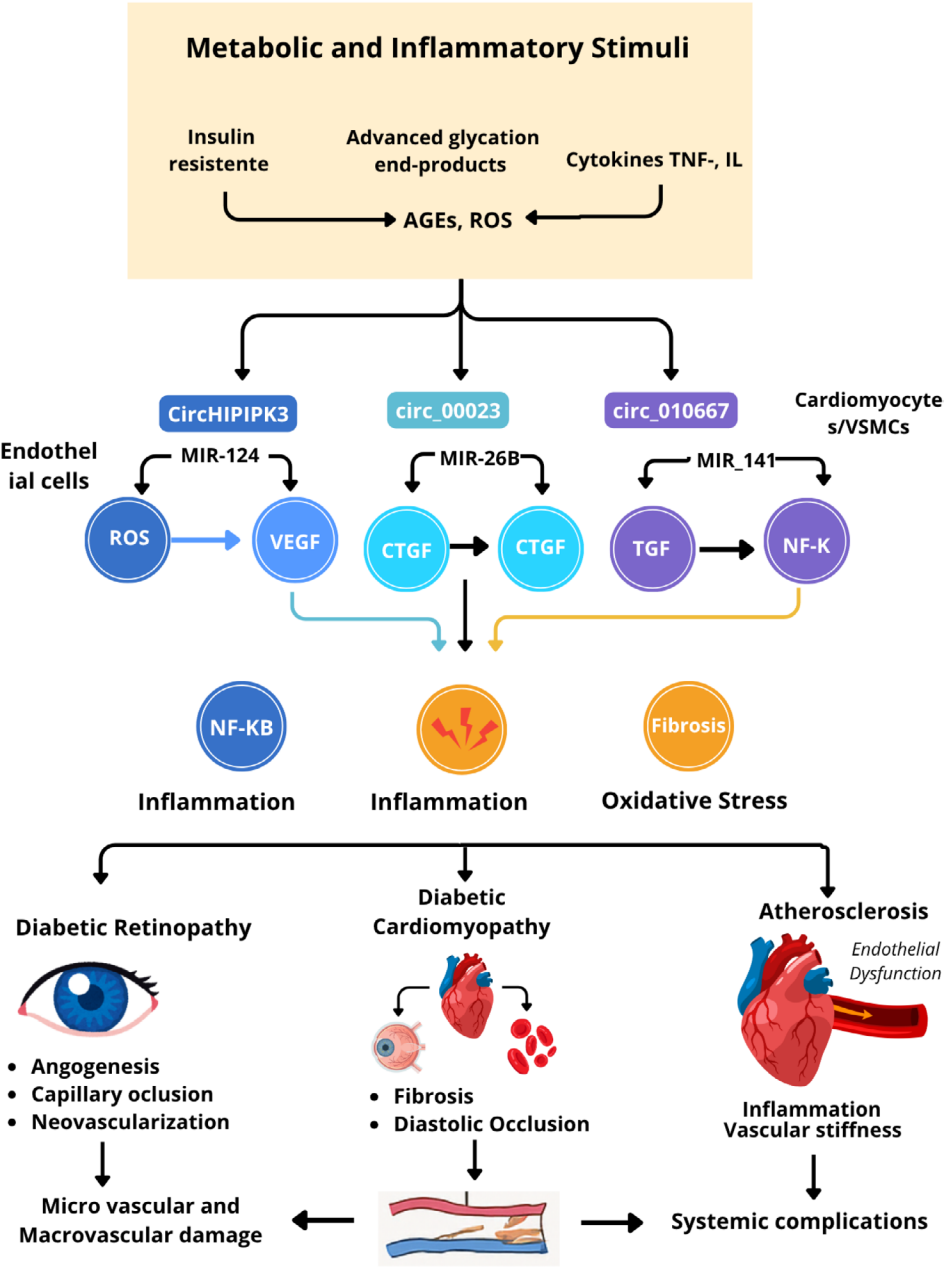
Integrative model of metabolic and inflammatory pathways linking circRNAs to diabetes‐related vascular and cardiac complications.

As shown in Table 1 and Figure 9, in diabetes, chronic exposure to hyperglycemia and free fatty acids promotes endothelial activation, inflammation and vascular stiffness, processes in which circRNAs are critically involved.

- circANKRD36 mediates inflammatory activation through hsa‐miR‐501‐5p, hsa‐miR‐3614‐3p and hsa‐miR‐498, upregulating proinflammatory cytokines [81].
- hsa_circ_0068087 promotes endothelial dysfunction by triggering the miR‐197/TLR4/NF‐κB/NLRP3 axis, linking innate immunity to vascular injury [82].
- hsa_circRNA11783‐2 has been associated with type 2 diabetes–related CAD, reinforcing its potential as a circulating biomarker of macrovascular complications [83].

**TABLE 1:** Summarising the role of different circular RNAs in the complications of diabetes.

CircCOL1A2	Diabetic retinopathy	Upregulated	Promotes angiogenesis	[54]
CircDNMT3B	Diabetic retinopathy	Upregulated	Reduces neoangiogenesis	[59]
hsa_circ_0041795	Diabetic retinopathy	Downregulated	Inhibits apoptosis	[61]
CircRNA_0084043	Diabetic retinopathy	Downregulated	Inhibits apoptosis	[62]
Circ‐ADAM9	Diabetic retinopathy	Upregulated	Promotes apoptosis	[63]
Circ‐PSEN1	Diabetic retinopathy	Downregulated	Ameliorates ferroptosis of RPE cells	[64]
CircRNA_010383	Diabetic nephropathy	Upregulated	Inhibit renal fibrosis	[65]
CircHIPK3 CircSMAD4 Circ‐FBXW12 Circ‐AKT3	Diabetic nephropathy	Downregulated Upregulated Downregulated Upregulated	Decrease fibrogenic factors Inhibits ECM deposition	[66, 67, 68, 69, 70]
Circ_0000712	Diabetic nephropathy	Upregulated	Inflammation and fibrosis	[71]
CircRNa.4614	Diabetic neuropathy	Upregulated	—	[75]
CircHIPK3	Diabetic neuropathy	Upregulated	Increase in neuropathic type of pain	[76]
CircRNA_000203 and CircHIPK3	Diabetic cardiomyopathy	Upregulated	Increase cardiac fibrosis	[77, 78, 79]
CircHIPK3	Diabetic cardiomyopathy	Upregulated	Protects cardiomyocytes	[80]
CircANKRD36	Cardiovascular disease	Upregulated	Pro‐inflammatory	[81]

Although several other circRNAs have been correlated with atherosclerosis and CAD, their specific contribution to diabetic vascular pathology remains under investigation [84].

Chronic metabolic stress induced by insulin resistance and advanced glycation end‐products (AGEs) activates inflammatory cytokines and reactive oxygen species (ROS), triggering the expression of specific circular RNAs (circHIPK3, circ_000203, circ_010667) in endothelial cells, vascular smooth muscle cells and cardiomyocytes. These circRNAs modulate key signalling intermediates including VEGF, CTGF, TGF‐β and NF‐κB, promoting oxidative stress, fibrosis and vascular inflammation. The downstream consequences include DR, DCM and atherosclerosis, culminating in systemic microvascular and macrovascular damage characteristic of DM [45, 50, 52, 77, 78, 79, 80, 83, 84].

### 5.7 The Therapeutic Effects of CircRNAs

**FIGURE 10:**
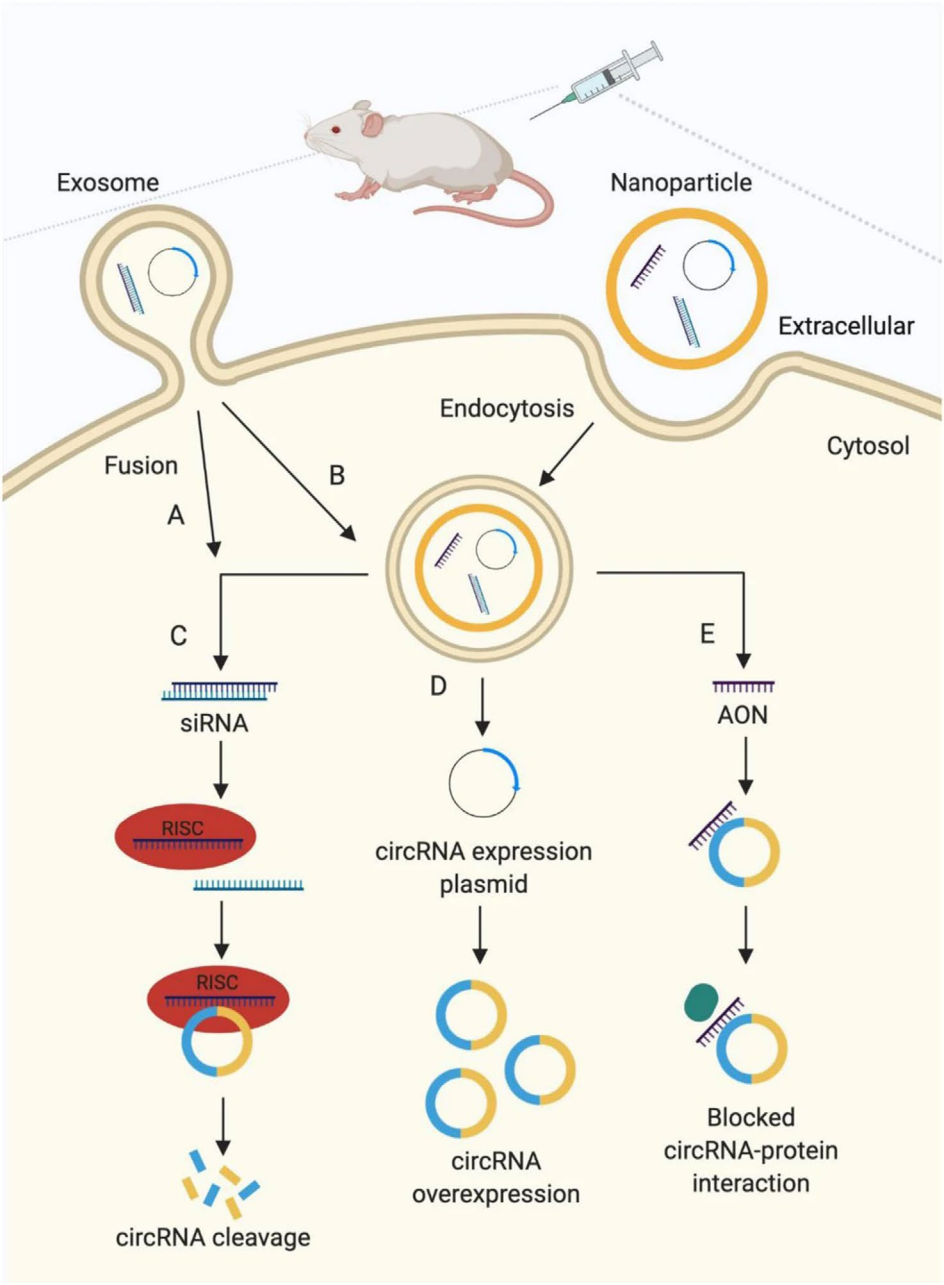
Strategies used to target circular RNAs (circRNAs) as a therapeutic approach in vivo. A—Exosome‐mediated delivery of A short interfering RNA (siRNA) targeting the back‐splice junction of circRNAs to induce circRNA cleavage and B—circRNA expression plasmid to overexpress circRNAs. (C–E)—Gold nanoparticle‐mediated delivery of C siRNA targeting the back‐splice junction of circRNAs, D—circRNA expression plasmid and E—antisense oligonucleotide (AON) blocking protein interaction site on circRNAs [91].

CircRNAs are expressed by at least 20% of currently active genes, according to high‐throughput RNA sequencing [85, 86]. Many unique circRNAs are produced per cell type, with circRNA isoforms of at least 3–10 different types created per host gene. Splicing creates circRNAs, and traditional splice sites—mostly located near annotated exon boundaries—are used for circularization [33]. Circularization is done by using splice sites backwards, whereby splice donors that are downstream are ‘backspliced’ to splice acceptors that are upstream. Most circRNAs are composed of 30–50 linked circles with exons and no intervening introns. The average length of circRNAs is 500 ribonucleotides (nts), with 1–4 exons [87]. There are few notable exceptions to the general rule that circRNAs are abundant in the cytoplasm. Four to six Exon‐Intron‐containing circular RNAs (EIciRNAs) are a class of circularization events in which one‐fifth of all circularization events include introns remaining in the circRNAs that have matured. Circular intronic RNAs (ciRNAs), a minor subset of circRNAs with just introns as well as no exons, originate from intronic lariats that have been normally spliced out rather than via backsplicing. The presence and purpose of ciRNAs and EIciRNAs in the nucleus define them [88]. All kinds of circular RNAs (circRNAs) are more stable than many linear RNAs due to their resistance to exonucleolytic degradation caused by the cellular exosome ribonuclease complex. Endogenously generated 30–50‐linked circRNA was shown to have an average half‐life of 19 to 24 h with a maximum half‐life of 48 h. On the other hand, the average lifetime of linear mRNAs is only 4 to 9 h. A key factor making circRNAs appealing for RNA‐centred medicinal applications is their high stability in biological systems [89, 90]. Figure 10 below shows the various therapeutic uses of circRNAs.

To investigate the function of noncoding RNA (ncRNA) in the pathophysiology of DM, several investigations were conducted. In DCM‐affected mice, elevated miRNA‐203 was shown to prevent oxidative stress and cardiac fibrosis [92]. DCM has also been shown to be substantially correlated with aberrant expression of lncRNAs [93]. It has been observed that in models of DCM, the long noncoding RNA (lncRNA) known as myocardial infarction‐associated transcript (MIAT) is expressed at an elevated level [94, 95]. Interestingly, silencing this transcript has been shown to improve cardiac function and reduce cardiomyocyte apoptosis. Recent research indicates that the myocardium of diabetic mice has elevated levels of circRNA 000203 [77]. Synthetic circular RNAs have been investigated as sponges of miRNA to treat CVDs. Adeno‐associated viruses (AAVs) were used in one instance for creating a circRNA that attaches cardiac pro‐hypertrophic miR‐212 and miR‐132 through 12 sites. After that, they were administered to an animal with aortic constriction to reduce hypertrophic cardiomyopathy [96]. Such circular RNAs inhibit miRNA function better than the benchmark antagonist antagomiRs, which are currently in use. The finding implies that myocardial fibrosis in DCM may be prevented and treated by targeting circRNA 000203 [97]. Table 2 below compares circRNAs with miRNAs and antisense oligonucleotides over various aspects.

**TABLE 2:** Comparative analysis of circRNAs, miRNAs and antisense oligonucleotides (ASOs) in diagnostics, therapeutics and stability.

Property	circRNAs	miRNAs	ASOs
Diagnostic use	Highly stable in blood/exosomes; tissue‐ and disease‐specific expression. Shown as effective biomarkers in heart disease (e.g., circ‐YOD1 in CAD, AUC≈0.82); also Ideal for noninvasive tests [98]	Circulating miRNAs (e.g., miR‐499, miR‐208b) are well‐known biomarkers in AMI and cancer [99]. Moderately stable if protected in exosomes. However, each miRNA is less tissue‐specific and can be altered in multiple conditions [100]	None established. ASOs are therapeutic molecules, not used as biomarkers
Therapeutic potential	Emerging modality. Can be used as stable RNA vaccines (cancer/viral) with improved stability and manufacturability. Multiple circRNAs are implicated in CVD and cancer (potential targets). No approved therapies yet [101]	Investigational. Strategies include miRNA mimics or inhibitors (antagomirs). Trials (e.g., MRX34 for cancer) have faced toxicity. No approved miRNA drugs; efficacy and safety challenges remain [102]	Well‐established. ASOs can knock down or modulate splicing of disease RNAs. Dozens of ASO drugs approved. Under development for many diseases [103]
Stability (half‐life)	Very high. CircRNAs resist RNases (no free ends); typical cellular half‐life > 48 h. This stability enables it to accumulate in cardiac cells. Long‐term stability in vivo and in vitro [100]	Moderate. Mature miRNAs in RISC or exosomes are stable, but free linear miRNAs degrade rapidly. Synthetic mimics require modification for stability	High (with modifications). PS‐backbone and 2′‐modifications render ASOs nuclease‐resistant, with tissue half‐lives of days–weeks [103]
Delivery methods	Usually via lipid nanoparticles or viral vectors (similar to mRNA vaccines). Can be injected or delivered to dendritic cells for vaccines. Experimental use of exosomes and hydrogels [101]	Delivered by LNPs or viral vectors for mimics/inhibitors or via conjugates (cholesterol, GalNAc) for specific tissues. Delivery remains a challenge [102]	Administered by injection (IV, SC, intrathecal). Can be delivered in LNPs or conjugated. Entry aided by PS backbone binding serum proteins [104]
Specificity	Moderate–high. CircRNA sequences (back‐splice junction) are unique, and expression is cell‐specific. However, circRNA ‘sponges’ may bind multiple miRNAs. Can design circRNAs for one target [100]	Low. miRNAs have short seed sequences (~6 nt) that match hundreds of genes. Each miRNA generally regulates many transcripts, so specificity is poor [105]	High. ASOs are designed for exact Watson–Crick binding to target RNA. Single‐base mismatches usually prevent binding. Very few off targets if the sequence is unique [103]

Abbreviations: AUC, area under the curve; CAD, coronary artery disease; LNP, lipid nanoparticles; RISC, RNA induced silencing complex.

It can be inferred from Table 2:

- CircRNAs have matched or even exceeded MiRNA in early detection of CVD, whereas ASO have no role in diagnosis.
- CircRNAs have a promising role in therapeutic effects considering their exceptional stability and intrinsic safety and show better promise than miRNAs, whereas ASOs have already been well established in therapeutics.
- CircRNAs are considered superior to miRNAs in terms of safety due to their innate exonuclease resistance, which gives them a longer half‐life in comparison to miRNAs.
- Both circRNAs and miRNAs benefit from delivery systems using LPNs and viral vectors. delivery technique is a common challenge for RNA‐based techniques.
- CircRNAs are specific by sequence, but they can be multifunctional, whereas miRNAs are the least specific since they target many genes. ASO leads in terms of specificity.

### 5.8 Challenges in CircRNA Research

Although circRNA‐based therapies hold immense promise, several technical and translational challenges remain before their clinical application can be realised. Most studies to date have been confined to preclinical models, and significant work is still required to ensure safety, specificity and efficacy in humans.

One of the primary obstacles is off‐target gene suppression associated with RNA interference (RNAi)–based approaches, where small interfering RNAs (siRNAs) may inadvertently silence unrelated genes through miRNA‐like mechanisms [106, 107, 108]. Emerging genome‐editing platforms such as CRISPR/Cas13 offer a more specific strategy for circRNA knockdown, with lower mismatch tolerance and enhanced selectivity, although their long‐term safety and efficiency in vivo remain to be demonstrated [109].

Another challenge involves immunogenicity and purity of synthetic circRNAs. Exogenously synthesised circRNAs can trigger innate immune responses due to the absence of m6A modifications, which distinguish them from endogenous transcripts [110]. To mitigate this, ongoing research explores chemical modifications, RBP coatings and optimised in vitro synthesis protocols that improve biocompatibility and molecular fidelity [111]. Furthermore, current circRNA overexpression systems rely on intronic complementarity, which may generate unwanted linear RNA byproducts or mis‐spliced intermediates, reducing yield and specificity. Producing high‐purity synthetic circRNAs on a large scale remains a technological bottleneck that limits their therapeutic scalability [112].

Delivery remains the most formidable challenge in circRNA therapeutics. Achieving targeted, efficient and safe delivery to specific tissues—particularly cardiac and endothelial cells—requires advanced carriers capable of crossing biological barriers while avoiding systemic toxicity. Nanoparticle‐based systems, including lipid nanoparticles and engineered exosomes, offer a promising solution by enhancing cell‐specific uptake and reducing off‐target exposure [113, 114, 115, 116]. These innovations, together with tissue‐selective promoters and chemical conjugation strategies, are expected to improve the precision and safety of circRNA modulation.

The rapid advancement of molecular detection technologies has also reshaped circRNA research. Quantitative RT‐PCR, droplet digital PCR, northern blotting and fluorescence in situ hybridization (FISH) have improved the sensitivity of circRNA quantification, while computational algorithms such as CIRCexplorer, find_circ and CIRI have enhanced detection from RNA‐sequencing data. Nevertheless, there is still no gold‐standard pipeline for circRNA identification, normalisation or annotation. As circRNAs perform diverse functions beyond miRNA sponging—such as regulation of transcription, mRNA stability and peptide translation—comprehensive functional studies in both physiological and pathological contexts are crucial to fully elucidate their biological roles [117, 118].

## 6 Conclusion

Cardiovascular complications account for most of the mortality associated with DM, despite advances in conventional therapies. The discovery of circRNAs has unveiled a new layer of gene regulation that contributes to the pathogenesis of these disorders through modulation of inflammation, oxidative stress, apoptosis and fibrosis. Their extraordinary stability, tissue specificity and presence in circulation make them attractive biomarkers for early diagnosis, while their regulatory versatility positions them as therapeutic targets for precision medicine.

Among the circRNAs implicated in diabetes‐related CVD, circ_000203 and circ_010567 have emerged as key modulators of myocardial fibrosis, whereas others such as circHIPK3 and circZNF609 participate in vascular remodelling and endothelial dysfunction. Early detection of these molecules may facilitate the identification of patients at higher risk for cardiovascular complications, allowing for timely preventive intervention.

Although circRNA‐based therapies remain in the preclinical phase, continuous progress in RNA nanotechnology, bioinformatics and CRISPR‐based editing brings them closer to translational application. A deeper understanding of circRNA networks and their molecular interactions will be essential for the development of safe, specific and clinically effective RNA therapeutics. As research advances, circRNAs are poised to become a cornerstone in the molecular diagnosis and personalised treatment of diabetes‐related CVD.

## Author Contributions

**Shreya Singh Beniwal:** conceptualization, literature search, data curation, primary manuscript drafting, and critical revision of the manuscript. **Yujin Jeong:** literature review, data interpretation, and contribution to manuscript development. **Akash Rawat:** literature search, data synthesis, and contribution to manuscript drafting. **Mahmoud Einieh:** critical analysis of the literature, interpretation of findings, and manuscript review and editing. **Kashyapi Patil:** data curation, assistance in manuscript drafting, and preparation of tables and schematic concepts. **Rafael Everton Assunção Ribeiro da Costa:** methodological guidance, validation of scientific content, and critical revision for intellectual rigor. **Pulkit Saini:** literature screening, contribution to manuscript drafting, and manuscript editing. **Kamal Yousef Ghazal:** clinical interpretation, contextual analysis, and critical revision of the manuscript. **Abhijeet Kumar:** data organization, reference management, and assistance in manuscript drafting and revision. **Ayush Dwivedi:** supervision, conceptual guidance, manuscript coordination, critical revision for intellectual content, and final approval of the version to be published. **Anuja Anil Mandavkar:** literature review, data interpretation, and contribution to manuscript revision and editing. All authors read and approved the final manuscript and agree to be accountable for all aspects of the work, ensuring accuracy and integrity.

## Conflicts of Interest

The authors declare no conflicts of interest.

## Data Availability

Data sharing not applicable to this article as no datasets were generated or analysed during the current study.
